# Role of 14-3-3σ in poor prognosis and in radiation and drug resistance of human pancreatic cancers

**DOI:** 10.1186/1471-2407-10-598

**Published:** 2010-11-01

**Authors:** Zhaomin Li, Zizheng Dong, David Myer, Michele Yip-Schneider, Jianguo Liu, Ping Cui, C Max Schmidt, Jian-Ting Zhang

**Affiliations:** 1 Department of Pharmacology and Toxicology, Indiana University School of Medicine, Indianapolis, IN 46202, USA; 2 Department of Surgery, Indiana University School of Medicine, Indianapolis, IN 46202, USA; 3 Department of Biochemistry & Molecular Biology, Indiana University School of Medicine, Indianapolis, IN 46202, USA; 4 IU Simon Cancer Center, Indiana University School of Medicine, Indianapolis, IN 46202, USA

## Abstract

**Background:**

Pancreatic cancer is the fourth leading cause of death in the US. Unlike other solid tumors such as testicular cancer which are now curable, more than 90% of pancreatic cancer patients die due to lack of response to therapy. Recently, the level of 14-3-3σ mRNA was found to be increased in pancreatic cancers and this increased expression may contribute to the failure in treatment of pancreatic cancers. In the present study, we tested this hypothesis.

**Methods:**

Western blot analysis was used to determine 14-3-3σ protein level in fresh frozen tissues and was correlated to clinical outcome. A stable cell line expressing 14-3-3σ was established and the effect of 14-3-3σ over-expression on cellular response to radiation and anticancer drugs were tested using SRB assay and clonogenic assays. Cell cycle distribution and apoptosis analyses were performed using propidium iodide staining and PARP cleavage assays.

**Results:**

We found that 14-3-3σ protein level was increased significantly in about 71% (17 of 24) of human pancreatic cancer tissues and that the 14-3-3σ protein level in cancers correlated with lymph node metastasis and poor prognosis. Furthermore, we demonstrated that over-expression of 14-3-3σ in a pancreatic cancer cell line caused resistance to γ-irradiation as well as anticancer drugs by causing resistance to treatment-induced apoptosis and G2/M arrest.

**Conclusion:**

The increased level of 14-3-3σ protein likely contributes to the poor clinical outcome of human pancreatic cancers by causing resistance to radiation and anticancer drugs. Thus, 14-3-3σ may serve as a prognosis marker predicting survival of pancreatic cancer patients and guide the clinical treatment of these patients.

## Background

14-3-3σ is a member of a highly conserved family of 14-3-3 proteins that are present in all eukaryotic organisms. There are 7 known human 14-3-3 isotypes (β, γ, ε, η, ζ, σ and τ/θ) [1-3] and they play important roles in many biological activities by binding to and altering the subcellular localization and/or stability of key molecules in various signaling cascades [4,5]. 14-3-3σ was originally characterized as a human mammary epithelium marker 1 [6] and later rediscovered as an important molecule for cell cycle checkpoint regulation [7,8].

Recently, proteomic profiling of a drug-selected breast cancer cell line MCF7/AdVp3000 showed that the expression of 14-3-3σ was up-regulated and its expression correlates with the drug resistance level of a series of drug resistant breast cancer cell lines [9]. Further investigation indicated that the elevated expression of 14-3-3σ causes resistance to anticancer drugs by resisting drug-induced apoptosis and inducing cell cycle arrest for repair of DNA damage [9,10].

Pancreatic cancer is the fourth leading cause of death in the US. Unlike some other cancers such as testicular cancer which are now curable, pancreatic cancer is incurable and more than 90% of pancreatic cancer patients die due to lack of treatment response. 14-3-3σ has been reported to be one of a number of genes that have increased expression at its mRNA level in pancreatic cancer tissues as identified by microarray profiling analyses [11-14]. Thus, it is possible that increased 14-3-3σ expression may contribute to the failure in treatment of pancreatic cancers. In this study, we tested the hypotheses that 14-3-3σ is up-regulated in pancreatic cancers at its protein level and its over-expression may contribute to the failure of treatment of pancreatic cancers by causing resistance to radiation and chemotherapy.

Using fresh-frozen tissues combined with Western blot analysis, we found that 14-3-3σ expression was indeed up-regulated in about 71% of human pancreatic cancer tissues examined and that the 14-3-3σ expression level correlated with lymph node metastasis, consistent with poor prognosis. Furthermore, over-expression of ectopic 14-3-3σ in a pancreatic cancer cell line caused resistance to γ-irradiation as well as anticancer drugs gemcitabine, mitoxantrone and Adriamycin by causing resistance to treatment-induced apoptosis and G2/M arrest.

## Methods

### Human tissues

All studies involving human subjects have been conducted in strict compliance with and approved by the Institutional Review Board (IRB) of Indiana University School of Medicine and its affiliated hospitals. All patients on this study signed informed written consent for collection of pancreatic tissues at the time of routine operation per the Indiana University Pancreas Lesion Tissue Fluid Bank (IUPLTFB) or IU/Lilly Tissue Bank protocol approved by IRB as described above. Surgical pathology was confirmed in all patients. Pancreatic cancer was staged according to the most current AJCC Guidelines. The matching normal tissues were from the farthest point (at least 20 mm) away from lesion (tumor). Tissue specimens were aliquoted and frozen immediately in liquid nitrogen after procurement and stored at -80°C.

### Sample preparation and Western blot analysis

Sample preparation and Western blot analysis were performed as previously described [15]. Briefly, the frozen tissues were thawed and homogenized in TNN-SDS buffer (50 mM Tris-HCl, pH value 7.5, 150 mM NaCl, 0.5% Nonidet P-40, 50 mM NaF, 1 mM sodium orthovanadate, 1 mM dithiothreitol, 0.1% SDS, and 2 mM phenylmethylsulfonyl fluoride) by using pellet pestle (Fisher scientific) and the lysates were clarified by centrifugation (12,000 g for 30 min at 4°C).

The tissue or cell lysates were separated by SDS-PAGE and transferred to a PVDF membrane followed by a 2-hr incubation in blocking solution (Tris- or PBS-buffered saline containing 5% nonfat dried milk and 0.1% Tween 20) and a 2-hr incubation with monoclonal antibody against 14-3-3σ (Upstate, NY. 1:500 dilution). The antibody reaction was detected by horseradish peroxidase-conjugated anti-mouse IgG antibody and visualized using ECL. The relative intensity of the protein on Western blot was measured using AlphaEaseFC Analysis tools (Alpha Innotech).

### Cell lines and stable transfection

BxPc-3 cells (ATCC) were grown in RPMI1640 (Cellgro) supplemented with 10% fetal bovine serum (Gibco) and 1% penicillin/streptomycin mixture (BioWhittaker). MiaPaCa-2 cells (ATCC) were maintained in DMEM supplemented with 10% fetal bovine serum, 2.5% donor equine serum (HyClone), and 1% penicillin/streptomycin mixture.

The cDNA of 14-3-3σ was engineered into pcDNA3.1(+) (Invitrogen). Transfection of this plasmid and its vector control into MiaPaCa-2 cells were performed using Lipofectamine (Invitrogen) and stable clones were selected using 1 mg/ml G418 (Invitrogen) as previously described [9,10]. The stable clones were maintained in complete DMEM supplemented with 200 μg/ml G418. For survival and apoptosis assays, G418 was removed and the cells were maintained in G418-free medium for one week prior to the assays.

Similarly, the stable siRNA knockdown BxPc-3 cells were generated as previously described [9,10]. Briefly, BxPc-3 cells were transfected with pSilencer-σ (14-3-3σ shRNA cloned into pSilencer 3.1-H1neo vector) or scrambled shRNA construct [9,10] using lipofectamine followed by selection first with 100 μg/ml G418 for 3 days and cultured for one week in complete medium in the absence of G418. The cells were then selected with 1 mg/ml G418 for 2 weeks. Individual clones were tested for 14-3-3σ knockdown and positive clones were propagated and maintained in complete RPMI1640 medium.

### Cytotoxicity assay

Cytotoxicity assay was performed as previously described using sulforhodamine B (SRB) colorimetric [16] and clonogenic [17] assays. For SRB assay, cells were seeded in 96-well plate at 5000 cells/well and cultured for 24 hours followed by treatment with anticancer drugs such as Adriamycin, mitoxantrone, gemcitabine or γ-irradiation and cultured continuously for 3 days. The culture medium was then aspirated, and the cells were fixed and stained by addition of 0.4% (w/v) SRB (Sigma) in 1% acetic acid solution followed by incubation at room temperature for 20 minutes. Free SRB was removed by washing cells with 1% acetic acid 3 times. The bound SRB was then solubilized with 10 mM Tris-base, and the OD_570 nm _was determined using a 96-well plate reader (MRX, Dynex Technologies).

For clonogenic assay, cells were seeded in 6-well plate at 100-200 cells/well and cultured for 24 hours followed by irradiation treatment. The cells were then cultured for two weeks before being subjected to fixation and staining with crystal violent (0.005% in 20% methanol) for 30 min. The colonies were counted manually. The radiation enhancement factor was calculated as the ratio of the mean inactivation dose of control cells divided by the mean inactivation dose of 14-3-3σ over-expression and knockdown cells as previously described [18]. A value greater or less than 1 indicates significant radio-sensitization or -resistance, respectively.

### Cell cycle analysis

Cell cycle analysis was performed as previously described [10]. Briefly, 5 × 10^5 ^cells were seeded in a 10-cm dish and cultured for 24 hours followed by treatments with γ-irradiation (5 Gy) or mitoxantrone (10 nM) and continuing culture for 24 hours (γ-irradiation) or 72 hours (mitoxantrone). The cells were then harvested, washed, and fixed using ethanol followed by staining with propidium iodide and FACS analysis. Cell cycle profiles were determined using CELL Quest and ModiFit programs.

### Statistical analysis

Data analyses were performed with GraphPad Prism (GraphPad Software Inc.) and Excel 2004 (Microsoft, Seattle, WA). Statistical associations between categorical factors were assessed using the Fisher exact test. Survival time was measured from the time of surgery until death or last follow-up (censor date was March 1, 2009). The association of categorical factors with survival was assessed using the Kaplan-Meier method and was analyzed using the log-rank test. Statistical significance was set at *p *value < 0.05.

## Results

### 14-3-3σ protein level is up-regulated in human pancreatic cancers

**Figure 1:**
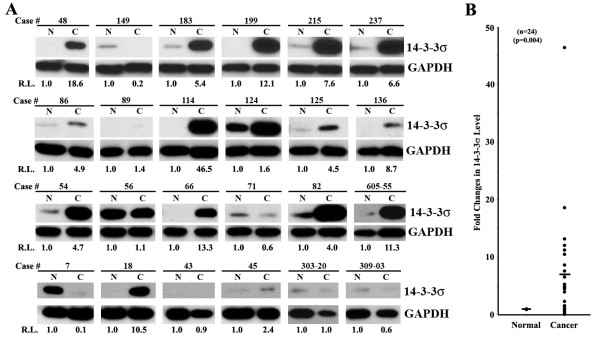
**14-3-3σ expression in paired normal and cancer pancreas**. A, Western blot analysis. Lysates from fresh frozen matching human normal (N) and pancreatic ductal adenocarcinoma (C) tissues were separated by SDS-PAGE followed by Western blot analysis of 14-3-3σ and GAPDH control. The relative level (R.L.) of 14-3-3σ in each sample was measured and normalized to that of GAPDH. The level of 14-3-3σ in each normal tissue was set to 1. B, summary of Western blot analysis. The relative levels of 14-3-3σ in normal and cancer tissues were graphed with the median level in each group marked by (-). Statistical analysis was done using Student T-test.

To determine the status of 14-3-3σ expression at its protein level in human pancreatic cancers relative to the corresponding normal tissues, we collected 24 pairs of fresh-frozen normal and cancer pancreatic tissues and determined the protein level of 14-3-3σ in these samples using Western blot. The relative 14-3-3σ protein level was then determined. As shown in Figure 1A, 17 of the 24 cases (70.8%) show increased 14-3-3σ expression in cancers compared to their matching normal pancreatic tissues. This increase is statistically significant (Figure 1B). In many cases, the increase is dramatic with one case (#114) demonstrating ~46-fold increase in cancer compared to normal tissues. Of the 24 cases, 4 (16.7%) (#149, #71, #7, #309-03) have decreased 14-3-3σ expression in cancer tissues whereas the remaining 3 (12.5%) (#56, #43, and #303-20) show no observable differences between normal and cancer tissues. These data clearly indicate that the 14-3-3σ protein level is significantly increased in the majority of pancreatic cancer tissues compared to their corresponding normal tissues.

### Up-regulated 14-3-3σ expression correlates with lymph node metastasis and mortality

**Figure 2:**
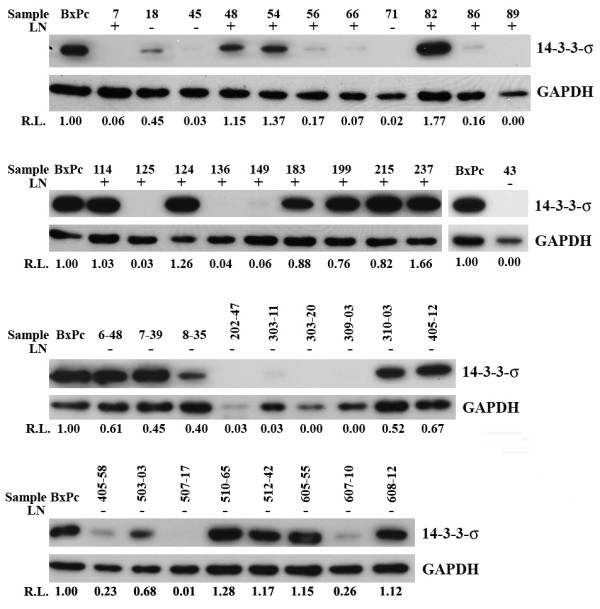
**Correlation of 14-3-3σ expression in pancreatic cancer tissues with lymph node metastasis**. Thirty-eight fresh frozen human pancreatic ductal adenocarcinoma tissues with (+) or without (-) lymph node (LN) metastasis were analyzed using Western blot for the protein level of 14-3-3σ and GAPDH control. The relative level (R.L.) of 14-3-3σ in each sample was measured and normalized to that of GAPDH. In each gel, lysates from BxPc-3 (BxPc) cells were used as a control and the level of 14-3-3σ in BxPc-3 cells was set to 1.

We next examined if the protein level of 14-3-3σ in pancreatic cancers correlates with lymph node metastasis, an indicator of poor prognosis. For this purpose, additional frozen tissues of pancreatic cancers were collected without matching normal tissues. These additional samples are 6-48, 7-39, 8-35, 202-47, 303-11, 310-03, 405-12, 405-58, 503-03, 507-17, 510-65, 512-42, 605-55, 607-10, and 608-12 (see Figure 2). This represents a total of 38 pancreatic cancer tissues of which 17 have lymph node metastasis and 21 do not (Table 1). The 14-3-3σ protein level was then determined using Western blot in all these cancer tissues relative to a known pancreatic cancer cell line, BxPc-3, which expresses endogenous 14-3-3σ (Figure 2). The relative expression level of 14-3-3σ in all cancer tissues was then correlated with the status of lymph node metastasis. As shown in Table 1, 52.9% of cancer tissues with lymph node metastasis whereas only 19.0% of cancer tissues without lymph node metastasis have high 14-3-3σ expression, a statistically significant difference (Table 1). Thus, higher expression level of 14-3-3σ correlates with lymph node metastasis.

**Table 1:** Correlation between 14-3-3σ expression and lymph node metastasis

Cancer Tissue	**14-3-3σ expression level**^**a**^		Total	**P value**^**b**^
			
	High	Low		
Lymph Node (+)	9 (52.9%)	8 (47.1%)	17	0.04
Lymph Node (-)	4 (19.0%)	17 (81.0%)	21	
Total	13	25	38	

^a^The expression level was designated high if the staining intensity is above 0.68 and low if equal to or below 0.68 in a post hoc analysis.

^b^Two-sided Fisher's exact test was used.

**Figure 3:**
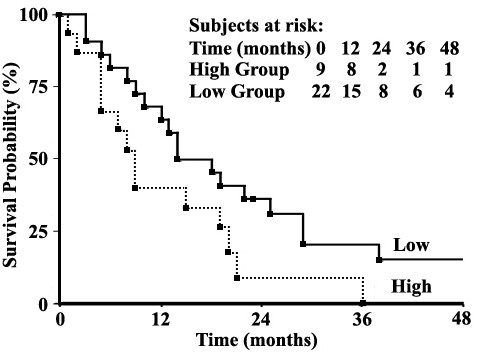
**Correlation of 14-3-3σ expression in pancreatic cancer tissues with patient survival**. Using Kaplan-Meier analysis, the high 14-3-3σ expression group has a trend of lower survival rate than the low expression group (*p *= 0.06, Log-Rank test).

Next, a correlation analysis between patient survival and 14-3-3σ protein level was performed. As shown in Figure 3, the Kaplan-Meier survival curves demonstrate a trend of higher survival for the low expression group compared to the high expression group (p = 0.06).

### Over-expression of 14-3-3σ causes resistance to radiation and anticancer drugs

**Figure 4:**
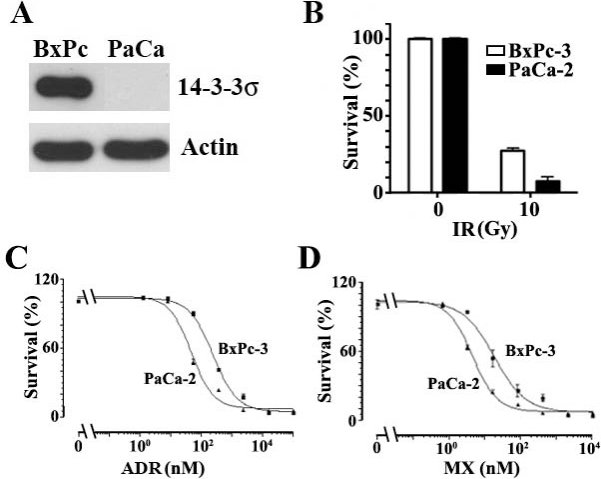
**Relationship between 14-3-3σ expression and drug resistance of pancreatic cancer cell lines**. A, Expression level of 14-3-3σ in BxPc-3 and MiaPaCa-2 cells. Lysates from BxPc-3 or MiaPaCa-2 cells were prepared for Western blot analyses of 14-3-3σ and actin as a loading control. B, C, and D, Correlation of 14-3-3σ expression and treatment resistance. The effect of γ-irradiation (B) and anticancer drugs Adriamycin (C) and mitoxantrone (D) on the survival of BxPc-3 and MiaPaCa-2 cells was determined using SRB assay. The survival following treatments was normalized to the control without irradiation or drug treatment.

To determine if 14-3-3σ expression is related to treatment resistance in pancreatic cancer, we first tested the treatment response of MiaPaCa-2, which does not express detectable level of 14-3-3σ, and BxPc-3, which expresses high levels of endogenous 14-3-3σ (Figure 4A). As shown in Figure 4B, BxPc-3 appears to be more resistant to γ-irradiation than MiaPaCa-2 cells, with more surviving cells after irradiation treatment. Figure 4C and 4D show that BxPc-3 cells are also much more resistant to anticancer drugs Adriamycin and mitoxantrone than MiaPaCa-2 cells. Clearly, the 14-3-3σ expression levels in these two pancreatic cancer cell lines positively correlate with their level of radiation and drug resistance.

**Figure 5:**
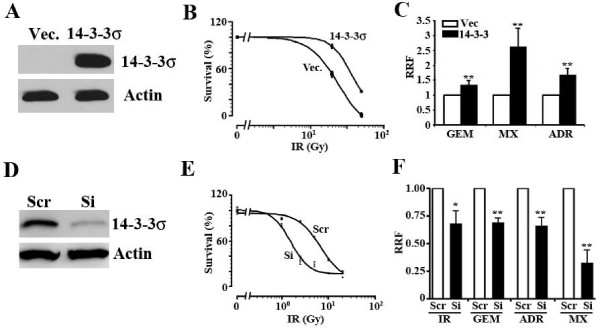
**14-3-3σ over-expression causes resistance to γ-irradiation and anticancer drugs in pancreatic cancer cell lines**. A, Western blot analysis. Lysates from MiaPaCa-2 cells stably transfected with 14-3-3σ or vector control were prepared for Western blot analyses of 14-3-3σ and actin as a control. B, Effect of 14-3-3σ over-expression on radiation resistance. Clonogenic assay was used to determine the response of MiaPaCa-2 stable clones transfected with 14-3-3σ or vector control (Vec.) to γ-irradiation (IR). C, Effect of 14-3-3σ over-expression on drug resistance. SRB assay was used to determine the response of MiaPaCa-2 stable clones transfected with 14-3-3σ or vector control (Vec.) to anticancer drugs gemcitabine (GEM), mitoxantrone (MX), and Adriamycin (Adr). RRF = relative resistance factor derived by dividing the IC_50 _of 14-3-3σ-expressing cells by that of the vector-transfected control cells. D, Western blot analysis. Lysates from BxPc-3 cells stably transfected with 14-3-3σ or scrambled shRNA control were prepared for Western blot analyses of 14-3-3σ and actin as a control. E and F, Effect of 14-3-3σ knockdown on treatment resistance. SRB assay was used to determine the response of BxPc-3 stable clone transfected with 14-3-3σ or scrambled (Scr) shRNA control to γ-irradiation and anticancer drugs. RRF = relative resistance factor derived by dividing the IC_50 _of 14-3-3σ-knockdown cells by that of the scrambled control cells. (* = p value < 0.05; ** = p value < 0.01).

To determine if 14-3-3σ expression causes drug resistance in pancreatic cancer cells, we established a stable MiaPaCa-2 cell line over-expressing ectopic 14-3-3σ (Figure 5A) and then tested its response to γ-irradiation first using clonogenic assay. As shown in Figure 5B, the stable 14-3-3σ-over-expressing cells appear to be more resistant to γ-irradiation than the vector-transfected (Vec) control cells with a radiation enhancement factor of 0.79. Thus, over-expression of 14-3-3σ in MiaPaCa-2 cells causes a significant increase in resistance to γ-irradiation.

We next investigated if over-expression of 14-3-3σ causes resistance to the anticancer drugs gemcitabine, mitoxantrone, and Adriamycin. As shown in Figure 5C, the stable 14-3-3σ-over-expressing cells are significantly more resistant to all three anticancer drugs compared with the stable vector-transfected control clone. Thus, ectopic over-expression of 14-3-3σ causes both radiation and drug resistance in MiaPaCa-2 cells.

To verify the above observations, we created a 14-3-3σ knockdown cell clone from BxPc-3 cells (Figure 5D) and tested if knocking down 14-3-3σ would decrease the resistance of BxPc-3 cells to radiation and drug treatment. As shown in Figure 5E and 5F, the stable clone with 14-3-3σ knockdown is significantly less resistant to γ-irradiation (with a radiation enhancement factor of 1.7), as well as to gemcitabine, Adriamycin, and mitoxantrone than the control cells transfected with scrambled control shRNAs. This finding further supports the conclusion that 14-3-3σ over-expression contributes to treatment resistance in pancreatic cancer cells.

### 14-3-3σ increases cell cycle arrest upon DNA damage

**Figure 6:**
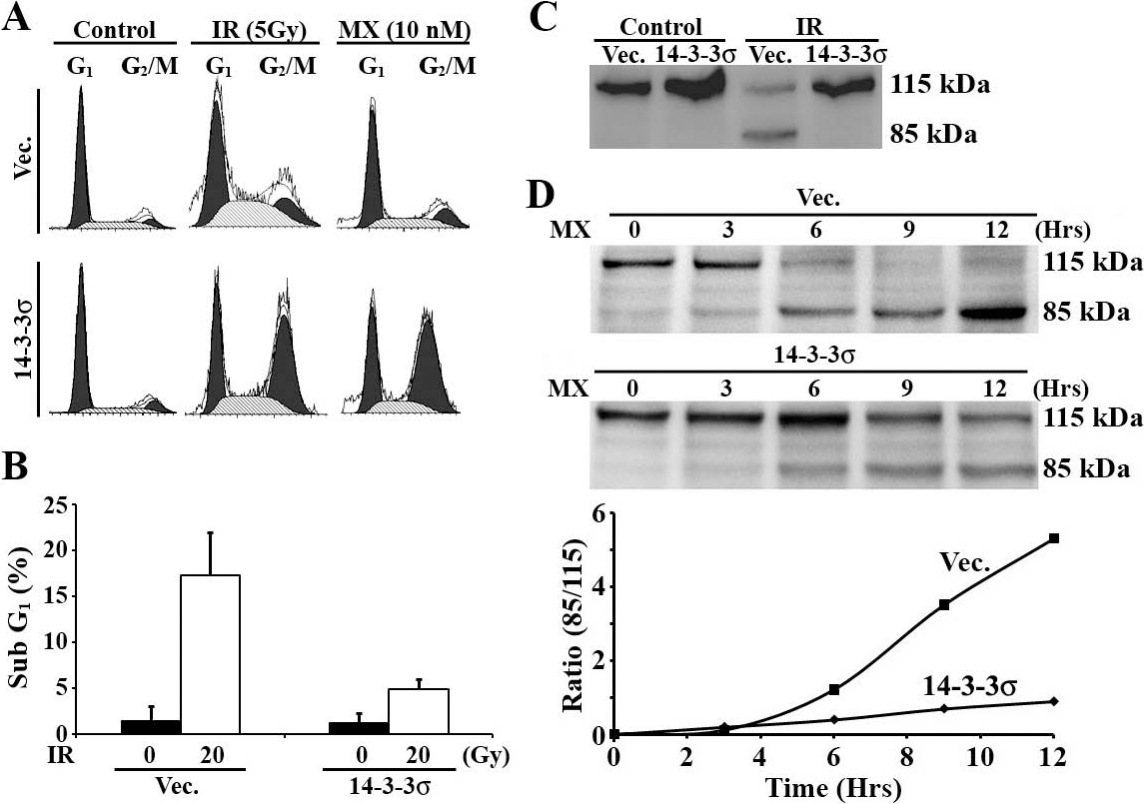
**14-3-3σ over-expression increased drug-induced G2/M arrest and resistance to drug-induced apoptosis**. A, Cell cycle distribution analysis. Vector- and 14-3-3σ-transfected stable MiaPaCa-2 cells were treated with 5 Gy γ-irradiation (IR), 10 nM mitoxantrone, or control without treatment followed by analysis of cell cycle distribution using FACS. B, Effect of 14-3-3σ radiation-induced apoptosis. Vector- and 14-3-3σ-transfected stable MiaPaCa-2 cells were treated with (filled bars) or without (white bars) γ-irradiation followed by analysis of cells at sub-G1 phase using FACS. C and D, Effect of 14-3-3σ on radiation- and drug-induced PARP cleavage. Vector- and 14-3-3σ-transfected stable MiaPaCa-2 cells were treated with 20 Gy γ-irradiation (C) or 10 μM mitoxantrone (D) for the times indicated followed by analysis of PARP cleavage using Western blot analysis. The graph in panel D shows the rate of production of the 85-kDa PARP fragments product (as a ratio of the 85-kDa fragment to the full-length 115-kDa protein).

Previously, we observed that over-expression of 14-3-3σ increases G2/M arrest upon DNA damage in prostate cancer cells as a mechanism of survival, by allowing more time to repair DNA damage [10]. To determine if the over-expression of 14-3-3σ also causes similar G2/M arrest upon DNA damage in pancreatic cancer cells, we treated the stable 14-3-3σ-over-expressing and vec-transfected control cells with γ-irradiation or mitoxantrone followed by FACS analysis of cell cycle distribution. As shown in Figure 6A, the stable 14-3-3σ-over-expressing cells clearly have a much higher proportion of cells arrested at G2/M than the vector-transfected control cells following γ-irradiation or mitoxantrone treatment. Thus, in pancreatic cancer cells 14-3-3σ likely provides a protection mechanism by arresting cells at G2/M phase, thereby providing cells an opportunity to repair DNA damage and survive DNA-damaging treatments.

### 14-3-3σ over-expression causes resistance to treatment-induced apoptosis

We next examined if the increased resistance of the stable 14-3-3σ-over-expressing cells was also due to inhibition of treatment-induced apoptosis. For this purpose, we first examined the population of stable 14-3-3σ-over-expressing and vector-transfected control cells at the subG1 phase following γ-irradiation. As shown in Figure 6B, the stable 14-3-3σ-over-expressing cells clearly have much fewer cells at the subG1 phase compared with the vector-transfected control cells following γ-irradiation.

We next examined the cleavage of PARP, a target substrate of activated caspases, following treatment with γ-irradiation or with the anticancer drug mitoxantrone. As shown in Figure 6C, the cleaved 85-kDa fragment of PARP is produced only in the control vector-transfected cells following γ-irradiation. Production of the 85-kDa fragment by γ-irradiation is apparently inhibited by over-expression of 14-3-3σ. Figure 6D shows that mitoxantrone-induced production of the 85-kDa fragment of PARP is also inhibited by 14-3-3σ over-expression in the stable 14-3-3σ-over-expressing cells compared with the vector-transfected control. Together, these findings suggest that over-expression of 14-3-3σ in pancreatic cancer cells likely causes resistance to DNA-damage induced apoptosis and, thus, resistance to radiation and drug treatments.

## Discussion

In this study, we demonstrated that the 14-3-3σ protein level was increased in a majority of pancreatic cancer tissues studied (~71%) compared to their corresponding normal tissues and that the 14-3-3σ protein level correlated with lymph node metastasis and patient survival. We also showed that the increased expression of 14-3-3σ correlated with poor cellular response to γ-irradiation and anticancer drugs and that stable over-expression of ectopic 14-3-3σ in a human pancreatic cancer cell line, MiaPaCa-2, significantly increased its resistance level to γ-irradiation as well as anticancer drugs in part by causing resistance to the apoptosis induced by the treatments and by arresting cells in G2/M phase.

It has been reported that 14-3-3σ expression is lost or decreased in human cancers of breast [19,20], liver [21], vulva [22], mouth [23], as well as neuroendocrine tumors [24], and small and non-small cell lung [25,26] cancers. This decrease is thought to be due to hypermethylation of the CpG islands of the 14-3-3σ gene [19,25,27,28]. However, recent studies revealed that the loss of 14-3-3σ expression in breast cancers is a sporadic event and that its expression is up-regulated in some breast tumors [29,30]. From these later studies, it appears that majority of breast cancer cells with basal/myoepithelial phenotype show 14-3-3σ expression whereas some of the breast cancer cells with luminal epithelial cell differentiation show decreased or loss of 14-3-3σ expression. Furthermore, it has also been reported that 14-3-3σ expression is increased in lung cancers [31], and head and neck squamous cell carcinomas [32].

In this study, we demonstrated that 14-3-3σ protein was increased significantly in the majority (17 of 24) of paired fresh-frozen pancreatic cancer tissues examined. Previously, it has also been observed that 14-3-3σ is one of the genes with increased expression at the mRNA level in pancreatic cancer tissues using microarray profiling or real time PCR [11-13,33]. In the study by Friess et al. [11], a microarray analysis of 5600 human genes in 8 human pancreatic ductal adenocarcinomas and 8 normal tissues showed that 14-3-3σ is one of 120 genes that had increased expression at the mRNA level in cancer compared to normal tissues. Similarly, Iacobuzio-Donahue et al. [13] reported that 14-3-3σ is one of the genes with increased expression of mRNA in a cDNA microarray profiling study of 17 infiltrating pancreatic cancer tissues compared with 5 normal pancreatic tissues. Using cell lines and methylation specific PCR analysis it was found that the increased expression of 14-3-3σ mRNA in pancreatic cancer cells may be due to hypomethylation of its promoter region. This later observation is interesting since 14-3-3σ in other cancers such as breast cancer is likely to be hypermethylated, resulting in silencing of its expression. Again in a third study, Logsdon et al. [12] used microarray analysis of 10 pancreatic cancer and 5 normal tissues and found 14-3-3σ among 188 genes that had increased mRNA expression in pancreatic cancer tissues. Using laser capture dissection and real time PCR, Neupane and Korc [33] compared the mRNA levels of all seven 14-3-3 genes in 3 normal and 5 pancreatic ductal adenocarcinoma tissues and found that 14-3-3σ is the only gene that has significantly higher expression in cancer tissues. However, all the above studies evaluated the expression of 14-3-3σ at its mRNA level. Since the mRNA level does not always predict the protein level, we performed the current study to determine 14-3-3σ protein expression in pancreatic cancers and found that the 14-3-3σ protein level is indeed significantly increased in pancreatic cancers. This observation is consistent with a previous study by Hustinx et al. [34]. Taken together, these studies clearly show that 14-3-3σ expression is increased at both its mRNA and protein levels in pancreatic cancer tissues and the increase may be due to decreased methylation of its promoter.

The finding that the 14-3-3σ protein level is increased in pancreatic cancers is interesting considering that increased 14-3-3σ expression has been shown to cause drug resistance in breast and prostate cancer cell lines [9,10]. Indeed, we showed that the increased 14-3-3σ expression in pancreatic cancers appears to correlate with lymph node metastasis, a marker of poor prognosis, and poor survival in a post hoc analysis. Furthermore, over-expression of ectopic 14-3-3σ in a pancreatic cancer cell line, MiaPaCa-2, caused resistance to anticancer drugs as well as to γ-irradiation while knocking down its expression in BxPc-3 cells reduced the resistance. Thus, the increased 14-3-3σ expression in pancreatic cancer cells may contribute to the failure in the treatment of human pancreatic cancers. Clearly, a more extensive prospective study with more patient samples is needed to further warrant this conclusion.

Recently, it has also been found that patients with breast carcinomas (both luminal or basal/myoepithelial phenotypes) that have higher cytoplasmic staining of 14-3-3σ also have shorter survival compared with patients whose tumors have lower 14-3-3σ staining [30]. In estrogen receptor positive tumors, the relationship between 14-3-3σ and poor prognosis is even more significant. This later finding is interesting because it has been shown previously that estrogen regulates the stability of 14-3-3σ by affecting its proteosome-mediated degradation [35]. In another study of cyclin B1 expression in breast carcinoma, Suzuki et al. also showed that the expression of cyclin B1 and 14-3-3σ have a positive correlation and predict a poor prognosis [36]. In a third study, it was suggested that 14-3-3σ expression is an independent prognostic marker for poor survival of colorectal cancer patients [37]. In prostate cancers, we previously found an increase in 14-3-3σ expression as tumor progresses [38]. Adenocarcinomas with high Gleason scores (>7) had significantly higher staining intensities and higher percentages of 14-3-3σ immunoreactive cells than adenocarcinomas with low scores (<7). Adenocarcinomas with lymph node metastases had higher percentages of 14-3-3σ expression compared with adenocarcinomas without lymph node metastases. Thus, over-expression of 14-3-3σ may cause resistance to therapies in various cancers. However, it is noteworthy that opposite observations have also been made in endometrial cancer [39], head and neck cancer [40], NSCLC [41], and nasopharyngeal carcinoma [42] where it was found that the absence or low expression level of 14-3-3σ predicts poor survival. The reason for the difference between these cancers is currently unknown.

Several popular anticancer drugs used for cancer therapy such as mitoxantrone and Adriamycin are topo II inhibitors which exert their anti-neoplastic effects in susceptible cells by inducing apoptosis, mainly through their ability to induce DNA double-strand breaks. Similarly, γ-irradiation also causes apoptosis via inducing double strand DNA breaks. However, radiation has also been suggested to induce cell death via mitotic catastrophe. In response to a DNA double-strand break, cancer cells undergo apoptosis or go into cell cycle arrest for repair of DNA which prevents replication of damaged DNA or aberrant mitosis which leads to mitotic catastrophe and apoptosis, thereby, attenuating the toxic effect of the treatments. 14-3-3σ has been shown to be essential for maintaining G2, G1, S cell cycle arrest following DNA damage by interacting with and negatively regulating cyclin dependent kinase Cdc2, CDK2 and CDK4, which are responsible for cell cycle progression, and prevent mitotic catastrophe and apoptosis [8,10,43]. Cells without 14-3-3σ are unable to arrest their cell cycle progression and will undergo mitotic catastrophe and apoptosis following DNA damage [43]. Furthermore, 14-3-3σ interacts and negatively regulates Bax and possibly BAD, two pro-apoptotic proteins that regulate the release of mitochondria apoptogenic factors [44]. Thus, increased 14-3-3σ expression in pancreatic cancer cells likely causes resistance to DNA damage-induced cell death.

Gemcitabine, a commonly used drug for pancreatic cancer treatment, does not cause DNA double strand breaks. However, incorporation of gemcitabine into DNA also causes damage to DNA by generating premature termination of DNA replication. The observation that over-expression of ectopic 14-3-3σ increases while knocking down its expression reduces resistance to gemcitabine, suggests that 14-3-3σ may also play a role in regulating repair of premature terminations.

## Conclusion

14-3-3σ expression is clearly up-regulated in the majority of pancreatic cancers and it may play an important role in pancreatic cancer cell response to drug and radiation treatments and, thus, likely contributes to the failure in treatment of pancreatic cancers. 14-3-3σ may serve as a prognosis marker predicting survival of pancreatic cancer patients and help physician make critical treatment decisions. Future studies targeting 14-3-3σ for development of chemosensitizers may help better treat pancreatic cancers in combinational therapy.

## Competing interests

The authors declare that they have no competing interests.

## Authors' contributions

ZL performed studies of cell lines. ZD performed studies with human tissues. DM, MYS, JL, and PC participated in the studies with either cell lines and/or human tissues. CMS participated in design and coordination of studies with human tissues. JTZ conceived of the study, participated in its design and coordination and drafted the manuscript. All authors read and approved the final manuscript.

## Pre-publication history

The pre-publication history for this paper can be accessed here:

http://www.biomedcentral.com/1471-2407/10/598/prepub
